# Supplementary material for: Are carbon emissions trading and green financial instruments synergistic? -Comprehensive quantitative research based on content analysis
Source: PLoS One. 2024 Mar 7;19(3):e0298601. doi: 10.1371/journal.pone.0298601 (PMC10919601; doi:10.1371/journal.pone.0298601)
Supplement: S1 Data — (ZIP) [file pone.0298601.s001.zip › Data/地级市Z_F工作报告.zip!/╔╜╢1⁄2/╬1⁄2╖╗╩╨/└┤╫╘╣1⁄2╓┌║┼í╛┬φ┐╦╩2╛▌═°í┐_2019.docx]

各位代表:

现在,我代表市人民政府向大会报告工作,请予审议,并请市政协委员和其他列席会议的同志提出意见。

一、2018年工作回顾

2018年是极不平凡的一年。一年来,市政府坚持以习近平新时代中国特色社会主义思想和党的十九大精神为指导,全面贯彻中央大政方针,坚决落实省委、省政府决策部署,在市委坚强领导下,团结带领全市人民,沉着应对一系列重大挑战和考验,拼搏进取,攻坚克难,较好地完成了市十七届人大二次会议确定的目标任务。

——经济实现平稳健康发展。全市地区生产总值增长6.5%,一般公共预算收入同口径增长6.5%,固定资产投资增长4.4%,社会消费品零售总额增长8.8%,进出口总额增长11.1%,居民消费价格上涨2.6%,城镇登记失业率控制在2.9%,省下达约束性指标全面完成。

——新旧动能转换全面起势。在全省率先组建新旧动能转换基金。PPP项目入库和签约落地数量全省第一。7个省重点项目、36个省优选项目、54个市级重点支撑项目完成年度投资计划。工业技改投资增长21.9%,16家企业列入国家、省级“两化”融合贯标试点,上云企业超过3000家。动力装备、现代农业等4个产业集群入选全省首批现代优势产业集群。“四新”经济增加值预计增长8%,高新技术产业产值占比预计达到41%,国内发明专利申请量、授权量分别增长33.6%和26.1%,新注册商标件数增长74.4%,新增山东名牌45件。潍坊获批建设国家创新型城市。潍坊(寿光)高新区被科技部列入新建国家高新区考察评审名单。潍柴动力获得第三届中国质量奖,“重型商用车动力总成关键技术及应用”荣获国家科技进步一等奖。

——乡村振兴战略扎实推进。以习近平总书记两次肯定“诸城模式”“潍坊模式”“寿光模式”为动力,编制实施乡村振兴战略规划和三年行动计划,启动实施“2221”工程。国家农业开放发展综合试验区获国务院批准设立,全国蔬菜质量标准中心正式揭牌,北京大学现代农业研究院完成主体工程,成功创建为农产品质量安全市,寒亭国家现代农业产业园通过验收。农业科技进步贡献率达到65%。新发展农民专业合作社1269家、家庭农场500家。整合市级涉农资金5.8亿元,设立乡村振兴“资金池”。开工建设田园综合体26个。乡村旅游渐成气候。成功承办2018全国农商互联暨精准扶贫产销对接大会。农村改厕三年任务全面完成。新建改造“四好农村路”1509.5公里。完成造林14.7万亩。农村“三清”工作扎实推进。全市集体经济收入3万元以下村基本消除。4个县市入围全国县域经济百强,2个镇进入全省十强。

——改革开放持续深化。全面展开机构改革,与市场经济相适应的体制机制逐步完善。扎实推进“三去一降一补”,在全省率先实行能耗等(减)量替代,率先开展工业企业效益综合评价,重点行业产能利用率稳定在80%以上。为企业提供政策性担保再担保融资112亿元,规模全省第一。深度推进“放管服”改革,公布市县两级“一次办好”事项清单,“3545”专项改革目标基本实现。“多证合一”改革扩大到“四十五证合一”,全市新登记市场主体和企业分别增长10.4%和26.5%。2726家规模以上企业完成规范化公司制改制。成为国家首批社会信用体系建设示范城市。推进高质量“双招双引”,对外开放取得新成效。签约省外过亿元项目233个、总投资1458亿元。出台《关于支持人才创新创业的若干政策》,引进高等教育机构院所24家、高层次人才3200人,吸纳大学本科以上毕业生1.5万人。潍坊综合保税区北区正式封关运行。风筝会、鲁台会、中日韩产业博览会成为对外开放重要平台。风筝会荣膺“改革开放40年中国十佳品牌节会”。成功举办首届潍坊发展大会。王伯祥同志荣获全国改革开放40周年改革先锋称号。

——城市功能不断提升。“四个城市”建设扎实推进,全市城镇化率达到61.75%。启动新一轮城市总体规划修编,完成中心城区空间布局发展规划、海岸带规划和南部生态涵养区及绿色产业规划编制。打通11条城区道路,开工10条下穿青银高速道路,潍县中路升级改造工程全面开工,东、南外环路建设完成年度计划。启动中心城区154公里未治理河道三年整治工程,畅通城市“微循环”34.2公里,改造城市“微空间”11.6万平方米。建设地下综合管廊8.05公里。海绵城市综合评价居全省首位。顺利通过国家园林城市复查。荣获首批山东省适宜人居环境奖。济青高速改扩建完成主体工程93%,南绕城高速(潍日高速连接线)完成立项审批,潍日高速全线通车。济青高铁建成通车,潍坊北站投入运营。潍莱高铁加快推进,京沪高铁二通道、机场迁建、轨道交通等前期工作取得新进展。峡山水库胶东地区调蓄战略水源地工程顺利实施,黄水东调二期工程开工建设。临朐抽水蓄能电站获批建设。“十三五”期间拟建所有特高压工程全面落地。

——三大攻坚战成效显著。多渠道化解处置不良资产,组建实收资本20.5亿元的鲁中金融资产管理公司,银行业不良贷款率低于全省平均水平1.2个百分点。安全生产事故起数、死亡人数分别下降29.6%和21.5%。扫黑除恶专项斗争成效显著。圆满完成上合组织青岛峰会服务保障任务。337个省定扶贫工作重点村全部摘帽,新识别及返贫人员全部兜底脱贫。取缔整改“散乱污”企业2440家。全市大气优良天数达到213天,PM2.5、PM10、二氧化硫分别改善10.2%、8%和26.1%。河长制持续深化,湖长制全面实行,湾长制启动实施。省控以上重点河流全面消除劣五类水质,近岸海域环境质量稳定向好。中央、省环保督察反馈问题扎实整改。

——民生质量稳步提高。民生支出占一般公共预算支出比重达到80.2%,城乡居民人均可支配收入分别增长7.6%和7.4%。新增城镇就业10.8万人,发放创业担保贷款13.2亿元,省级创业型城市和镇街创建数量居全省第一。开工棚改项目7.03万套,改造农村危房1535户。高标准完成教育“全面改薄”任务,新增中小学、幼儿园学位4.8万个和3.5万个,基本消除大班额,国家义务教育优质均衡县创建走在全省前列。入选全省首批健康城市试点,被确定为国家公立医院薪酬制度改革试点城市、国家医养结合示范省先行区、国家中医药综合改革示范区、国家社会心理服务体系建设试点市。医改考核、人口计生考核均居全省第一。成为全国居家和社区养老服务改革试点地区。省级食品安全先进县实现全域创建。成功创评为第三届文化强省建设先进市。基层综合性文化服务中心达标率100%。行政村(社区)体育健身设施覆盖率达到96%以上。第二次全国地名普查圆满结束。国防动员和后备力量建设水平不断提升,军政军民团结更加巩固。妇女儿童权益保障进一步加强。老龄、残疾人事业迈上新台阶。外事侨务、民族宗教、海事、海渔、人防、地震、气象、史志、档案等工作取得新进展。

——抗灾救灾取得决定性胜利。去年,受“安比”“摩羯”“温比亚”台风叠加影响,潍坊遭遇1951年有气象记录以来最强降雨,全市11个县市区、101个镇街、157.9万人受灾,直接经济损失189亿元。灾情发生后,党中央、国务院和省委、省政府高度重视。习近平总书记专门作出重要批示,为全面夺取抗灾救灾胜利指明了方向。省委、省政府第一时间成立抗灾救灾指挥部和前线指挥部,主要领导同志多次亲临一线坐镇指挥。国家和省直有关部门单位给予大力支持、无私支援。国家应急管理部紧急调集山东、河北、江苏、天津4个省市的1万多名消防官兵、500多部消防车辆连夜驰援灾区；国家防总、民政部、农业农村部、中国疾控中心等国家部委(单位)和省直有关部门及时奔赴灾区,给予专业指导,下拨救灾款物；驻鲁、驻潍部队第一时间请战参战；各级各类媒体深入抗灾救灾一线,作了大量客观公正、生动深入的报道。兄弟地市、驻外商会及海内外朋友纷纷伸出援助之手,许多企业和群众自发到灾区送饭送物,以社会组织和教师学生为主体的志愿者靠在一线参与救灾,社会各界通过慈善总会、红十字会累计捐款捐物2.8亿元。对总书记的关心、各级的支持、社会各界的帮助,潍坊人民将永远铭记!

面对突如其来的灾情,市委、市政府把抗灾救灾作为压倒一切的任务,展开了潍坊历史上动员范围最广、投入力量最大的抗洪抢险斗争。全市上下团结一心、众志成城,基层党员干部舍生忘死、冲锋在前,广大群众自强自立、奋起自救,共同谱写了抗灾救灾的壮丽篇章。在这场艰苦卓绝的斗争中,我们十几个小时成功转移17.8万名群众,没有发生一起因转移不及时而导致的伤亡事故；在洪水过后,我们紧急妥善安置6.7万名受灾群众,保障了每名群众有饭吃、有衣穿、有干净水喝、有安全住处、有病能及时医治；我们调集专业力量,对灾区水利设施、房屋建筑、桥梁道路、电力设施、工业企业、地质灾害隐患等进行拉网式排查,对受灾房屋逐户进行检查鉴定,有效杜绝了次生灾害发生；我们快速抢修抢通,在最短时间内实现了水电路讯“四通”,所有灾区学校全部如期开学；我们全面加强灾区卫生防疫,清理垃圾、淤泥,开展大规模消杀,及时无害化处理死亡畜禽,做到了大灾之后零疫情；我们突击打好大棚排水除涝攻坚战,在省委书记刘家义同志的亲自指挥下,2天开挖疏通排水干渠75.8公里,8天完成排水3280万方,相当于三个西湖的水量,比预定时间提前约两个半月,挽回直接经济损失46亿元；我们及时调运救灾应急物资,启动政府市场价格稳定基金,政企联手平抑物价,确保了全市生活必需品市场的充足供应和价格稳定；我们积极建立保险理赔和金融机构联动投入机制,实施灾后重建贷款贴息政策,积极开展贷款发放和保险理赔,为灾后重建提供了有力的资金保障。

在抓好抢险救灾的同时,同步推进灾后重建和灾区群众安全温暖过冬工作。市委、市政府科学制定灾后重建总体方案及配套工作方案,研究确立“一四六三一”灾后重建工作思路。坚持把灾后群众生活保障和冬春生活救助作为头等大事,对全市需冬春救助的130658名受灾群众全部建立工作台账,对分散安置的8540名受灾群众每户发放联系卡。全面落实省委、省政府“8+7”政策措施,筹集拨付各类资金26.08亿元,保障灾后重建顺利推进。全市3948户一般受损房屋完成修缮加固,3966户倒损房屋完成分散重建,15个整体迁建村庄全部开工建设,因灾受损的6.8万个大棚完成加固整修,因灾倒塌的3.05万个大棚完成重建2.54万个,727家受灾企业全面恢复生产经营,总投资35亿元的398项水利设施重建项目按进度加快建设,国省道灾后重建工作全面完成,农村公路灾后重建完成过半,384所受灾学校、幼儿园已完成加固修缮380所,406处受损市政公用设施全面恢复。

在这场波澜壮阔的抗灾救灾斗争中,寿光市孙家集派出所孙超、魏泽坤两名年轻同志,为救援被洪水围困的群众英勇牺牲,献出了宝贵的生命。受灾县市的各级干部,尤其是最基层的广大党员干部群众,用初心凝聚力量、用使命铸就坚强,涌现出许多感天动地的英雄事迹,谱写了可歌可泣的时代壮歌。这段抗灾历程,将永载潍坊史册!这其中蕴含的抗灾精神,将激励我们奋勇前行!

——政府自身建设进一步加强。认真执行市人大及其常委会决议决定,自觉把民主政治协商纳入决策程序,办理人大代表议案建议169件、政协委员提案372件,提请市人大常委会审议地方性法规草案1件。完成建市以来政府文件清理工作。探索形成市区行政复议“一体化”办案模式。法治政府建设评估连续三年位居全国前列。政务公开第三方评估成绩和综合考核继续保持全省领先。便民服务中心和网上政务服务厅实现镇街全覆盖。2018中国“互联网+”数字政务指数年度增速全国第一,市政府网站绩效评估全省第一。“大学习、大调研、大改进”和“作风建设年”活动深入开展。创新推进领导干部自然资源资产审计,审计监督成效全面提升。政府系统党风廉政建设和反腐败工作扎实推进。

各位代表,一年来取得的成绩,是以习近平同志为核心的党中央举旗定向、坚强领导的结果,是省委、省政府科学决策、悉心指导的结果,是市委团结带领全市人民开拓进取、创新实干的结果,是市人大、市政协和社会各界监督支持、协同奋斗的结果。在此,我代表潍坊市人民政府,向全市人民、各界人士,向驻潍部队、武警官兵和公安干警,向中央、省属驻潍单位,向所有关心支持潍坊发展的海内外朋友们,表示衷心的感谢并致以崇高的敬意!

肯定成绩的同时,也要清醒看到,我市推进高质量发展还面临不少困难和问题。主要表现在:发展观念、发展理念与新时代要求还存在一定差距；产业结构偏重,产业层次不高,经济增长受要素制约较大；现代服务业发展不快,总量偏小；城市功能布局不够完善,中心城市带动力不强；城市管理不够精细,交通拥堵等“城市病”没有大的改善；大气、水等环境问题突出,环保压力较大；部分县市区债务率偏高,财政收支压力增大；教育、医疗、居住、养老等民生工作仍存在短板；政府工作人员法治思维、专业素养、合作精神还有欠缺,担当作为意识有待全面提升。需要特别说明的是,因遭受特大暴雨灾害,农村居民人均可支配收入增速低于年初计划；受多种因素影响,中心城区6家企业搬迁没有完成年度任务,反映出政府抓落实还需要加强。对发展中的问题、没有完成的任务,我们一定高度重视,采取有力措施,认真加以解决。

二、2019年经济社会发展的目标任务

今年是新中国成立70周年,是全面建成小康社会的关键之年。政府工作的总体要求是:以习近平新时代中国特色社会主义思想为指导,认真贯彻党的十九大和十九届二中、三中全会以及中央经济工作会议精神,深入落实习近平总书记视察山东重要讲话、重要指示批示精神,按照全省经济工作会议部署和市委“六三七五一”工作思路,坚持稳中求进工作总基调,坚持新发展理念,坚持高质量发展,坚持以供给侧结构性改革为主线,坚持深化市场化改革、扩大高水平开放,以新旧动能转换重大工程为引领,加大资源环境倒逼力度,加快实施创新驱动发展战略,聚焦聚力推进乡村振兴等重点工作,继续打好三大攻坚战,统筹推进稳增长、促改革、调结构、惠民生、防风险工作,保持经济运行在合理区间,进一步稳就业、稳金融、稳外贸、稳外资、稳投资、稳预期,着力激发微观主体活力,加快塑造高质量发展新优势,促进经济持续健康发展和社会和谐稳定,以优异成绩庆祝中华人民共和国成立70周年。

今年全市经济社会发展主要预期目标是:地区生产总值增长6.5%左右,固定资产投资、社会消费品零售总额增速与全省持平,进出口增速高于全省1.5个百分点以上,规模以上工业增加值增长5.5%左右,一般公共预算收入增长5%左右,城镇登记失业率控制在3.8%以内,城镇居民人均可支配收入增长7%,农村居民人均可支配收入增长7%以上,居民消费价格涨幅3%左右,全面完成省下达的节能减排降碳约束性指标和环境质量改善目标。

今年经济社会发展仍然面临复杂形势,同时也蕴含着重大机遇,这对政府工作提出了新的更高要求。我们将牢牢把握加强政治建设这个根本性建设。旗帜鲜明讲政治,坚持党的全面领导,树牢“四个意识”,坚定“四个自信”,坚决做到“四个服从”“两个维护”,自觉用习近平新时代中国特色社会主义思想武装头脑、指导实践、推动工作。牢牢把握市委“六三七五一”工作思路。深化“六个认识”,深化对做好新形势下经济工作“五大规律性”的认识,深化对我国发展重要战略机遇期的认识,深化对推动经济高质量发展的认识,深化对供给侧结构性改革的认识,深化对推进市场化改革、扩大高水平开放的认识,深化对提高党领导经济工作能力和水平的认识。用好“三个重大机遇”,用好支持民营经济发展的大好机遇,用好国家推动制造业高质量发展的重大机遇,用好习近平总书记充分肯定“诸城模式”“潍坊模式”“寿光模式”和建设国家农业开放发展综合试验区的重大机遇。把握“七个更加注重”,更加注重稳中求进,更加注重践行新发展理念,更加注重问题导向,更加注重求真务实,更加注重项目建设,更加注重全域统筹,更加注重防风险、守底线。突出“五项重点任务”,更大力度推进新旧动能转换,更深层次实施乡村振兴战略,更高水平推进改革开放,更加注重打好三大攻坚战,更加关注保障改善民生。扭住“一个工作抓手”,以“转观念、正风纪、夯基础、提质量、促发展”为主题,扎实开展“工作落实年”活动,确保各项目标任务落实落地。

围绕上述目标要求,重点做好以下六个方面工作:

(一)加快新旧动能转换,着力推动经济高质量发展。高质量发展,核心是高质量,关键是发展,根本措施是加快新旧动能转换。要坚定不移贯彻新发展理念,以供给侧结构性改革为主线,坚持质量第一、效益优先,准确把握“巩固、增强、提升、畅通”要求,主动转变发展方式,大力优化产业结构,加快转换增长动力,推动全市经济由规模数量型向质量效益型跃升转变。

以制造业为突破口,厚植产业竞争优势。制造业是潍坊的当家产业,发展制造业是我们的看家本领。要突出其战略地位,紧紧扭住不放,持续加力突破。聚焦重点产业。全面实施高端装备、汽车制造、高端化工等7个产业发展专项规划,深化纺织服装、食品加工、造纸包装3个产业集群转型提升方案,落实铸造行业转型升级实施意见和数字经济发展规划,强化要素资源保障,推动制造业高端化、智能化、品牌化发展。加快转型升级。深入开展企业技术改造、智能化改造、绿色化改造三年专项行动,强力实施800个重点技改项目,年内完成投资600亿元。新增省级以上“两化”融合贯标试点10个以上,培育10家智能制造标杆企业,培训企业首席信息官500人次。推动移动互联网应用由消费领域向生产领域拓展,积极发展智能化生产、协同化制造、网络化研发和个性化服务。扎实开展工业企业效益综合评价,建设市级综合评价数据分析平台,完善配套政策,加快淘汰落后产能,倒逼企业转型升级。推进集群发展。完善产业发展规划,重点打造国际动力城、高端化工产业基地、虚拟现实产业基地。全力支持潍柴动力产业园建设,集中培育和发展发动机、新能源、整机整车三大板块,加快形成万亿级企业。聚焦打造高端化工产业基地,加快化工产业转型升级,抓好弘润石化、昌邑石化、鲁清石化、新和成等重点园区、重点项目建设,进一步提升全市石化产业发展整体水平。加快歌尔智慧城等重点项目建设,进一步提升虚拟现实产业整体竞争力。加快生物基材料、新能源汽车、智能农机、新松智慧园等优势产业集聚区建设,创建一批省级“雁阵型”产业集群。支持主营业务收入过百亿的龙头企业扩张提质、加快发展,建设10个以上高端配套产业园区,加快建设智能农机装备生产基地、环渤海高端装备制造产业基地。壮大“两群”规模。深入实施龙头企业群、“隐形冠军”企业群培育方案,建立“两群”企业梯次培育名录。完善专项扶持政策,引导企业做好市场拓展、质量提升、品牌培育等工作。年内新增省长质量奖1个以上,新认定市级“隐形冠军”企业40家以上。

以补短板为重点,加快产业结构调整。聚焦产业层次偏低、产业结构偏重短板,坚持市场调节和政府引导相结合,以“四新”“四化”为目标,推动产业提档升级。加速发展新兴产业。实施新兴产业倍增工程,支持发展个性化定制、云制造、分享经济等新业态新模式。大力发展新一代信息技术、高端装备、新能源新材料等战略性新兴产业,加快建设激光雷达产业基地、青科大大数据学院等重点项目,培育壮大北斗导航应用产业链。年内形成10个以上高端装备产业集聚区、10个以上特色新材料产业基地,全市新兴产业增加值增长10%以上。加快突破服务业。在繁荣发展生活性服务业的同时,更加重视发展生产性服务业,推动先进制造业与现代服务业全面融合,提升制造服务化水平。大力发展现代物流业,抓好潍坊北站高铁物流园、滨海临港物流园、鲁东物流中心等重点园区建设,力争全市3A级以上物流企业达到40家以上。深入推进金融创新发展三年行动计划,加快建设金融创新中心和区域资本中心,促进现代金融与实体经济协同发展。进一步提升文化产业示范园区(基地)建设水平,培强做大一批文化龙头企业。推进文化与旅游深度融合,组建市文化旅游发展集团。实施文化旅游项目“双百”工程,策划打造潍坊全域旅游精品线路。依托八喜旅游网等电商平台,开展“百万人游潍坊”“潍坊旅游商品云展汇”活动。健全完善电子商务公共服务体系,推动传统商贸流通企业跨界融合,完成中心城区商品流通市场整治提升。完善会展经济激励政策,坚持市场化办会方向,提升风筝会、鲁台会、菜博会、中日韩产业博览会办会效益和层次。培育引进高端服务组织,大力发展服务贸易,支持各类中介服务业做强做大。加快发展医养健康产业,实施“全域医养健康城”工程,按照“一区两带五中心”产业布局,抓好潍坊生物医药产业园、潍坊奥精健康科技产业园、北辰医养健康产业园等20个医养健康重点园区建设,引进一批“医养康保”综合项目,打造国家医养结合示范省先行区。大力发展海洋经济。实施海洋强市建设“八大工程”,创新面向涉海企业的“海洋+”发展模式。加快56个海洋强省重点项目和100个市级海洋经济项目建设。率先突破海洋化工、海工装备、海洋新能源新材料等“拳头”产业。推进现代化海洋牧场综合试点,加快建设“海上粮仓”。积极创建国家“智慧海洋”工程建设示范区。推动军民融合发展。建立军地一体协同推进体系,支持引导具备条件的企业进入军工生产领域。加快推进军民融合通用航空产业园、高端装备制造军民融合产业园、山东航天威能新能源电池系统等重点项目,高标准建设省级军民融合示范基地和市级特色产业园。突出重点项目建设。加快推进9个省重点项目、36个省优选项目和180个市级重大重点项目建设。完善项目考核评价体系,落实领导包靠、问题直报、协调会商等工作机制,抓实集中开工、进度比武、观摩点评等专项活动,形成“大抓项目、抓大项目”的鲜明导向。

以改革创新为引领,全面增强产业发展内生动力。“惟改革者进,惟创新者强,惟改革创新者胜。”要坚持市场化改革与制造业技术创新双向发力,全面提升产业核心竞争能力。深化重点领域改革。扎实推进承担的国家和省改革试点任务,进一步放大试点效应。优化市以下财政体制,健全预算绩效管理机制。加快新旧动能转换基金运作,新设立并购基金、纾困基金。继续实施规模以上企业规范化公司制改制,年内力争2家企业在境内外主板首发上市、100家企业在“新三板”和区域性股权交易市场挂牌。发挥潍坊五板资本市场服务功能,年内入场企业达到5000家。推进要素市场化配置改革,深化“弹性年期”“租让结合”“长期租赁”等工业用地制度改革,抓好能源消费总量管理和煤炭消费控制。优化国有资本布局,加快整合重组步伐,重点投向基础设施、民生领域和新旧动能转换“十大产业”。深入推进混合所有制改革,实现国有资本与非国有资本融合发展。大力实施创新驱动发展战略。进一步加大创新投入,全社会研发投入占比提高到2.67%。强化创新平台建设,新建30家市级、10家省级以上科技创新平台,加快市工业研究院建设,新成立3家行业技术研究分院,规划建设市产业技术研究院,广泛吸引天使投资、创业投资、私募股权投资等参与,打造“政产学研金服用”创新共同体。强化普惠性、精准性政策供给,发挥好潍坊高新区龙头带动作用,支持潍柴国家燃料电池技术创新中心、国家智能铸造产业创新(潍坊)中心、峡山省级农业科技园区、潍坊(寿光)高新区建设。实施高新技术企业“育苗造林”行动计划,重视发挥民营高新技术企业在创新方面的优势和作用,新增高新技术企业50家,高新技术产业产值占比提高1个百分点。落实政府采购、首台套政策、强制性标准等政策工具,涵养和支撑自主创新能力。加快科技成果转移转化,建立健全以创新能力、创新质量、实际共享为导向的多元化激励机制,新认定市级技术转移机构5家以上,转化科技成果100项。提升科技合作交流水平,发挥好中以(潍坊)科技转移平台、潍坊(美国)硅谷高科技孵化器等国际化创新平台作用,在欧美、澳新、日韩等地建设6家海外研发中心。全面推进知识产权强市创建,建设运营好中国(潍坊)知识产权保护中心。

以“双招双引”为载体,加快打造对外开放新高地。进一步强化开放理念,自觉把潍坊发展放到全国、放到世界发展的大坐标大格局中定位,统筹国际国内两个市场、两种资源,构建全方位、多层次、宽领域开放发展新格局。精准推进“双招双引”。围绕壮大产业链抓招商,围绕培育新动能引人才,吸引更多创新型、基地型、补链型、平台型、终端型大项目落户潍坊。实施“一把手”招商工程,落实“一部门一招商项目”计划,实现招商和项目落地分级负责、一体推进。完善专业招商机制,建立高素质、复合型、专业化招商队伍,全面推行“招商合伙人”制度,完善代理招商网络。聚焦“一带一路”沿线国家、金砖国家、上合国家等重点国家和地区,务实开展系列招商活动。坚持人才引领发展的战略定位,大力实施人才强市战略,聚焦引得进、用得好、留得住,拓宽企业、高校、科研院所精准对接渠道,抓好国家“千人计划”、泰山系列和鸢都系列重点人才工程,举办中国(潍坊)创新创业大赛,强化信息化、柔性化、常态化招才引智。建立鸢都惠才卡制度,落实人才专员服务清单,规划建设一批仿真国际人才社区、国际学校、国际门诊等,优化人才生态环境。全面扩大对外开放。实施“千企百展”国际市场开拓计划,支持企业整合全球资源,深度开拓重点市场,加快出口市场多元化。完善跨境电商、外贸综合服务平台等新业态支持政策,积极推行“一次申报、一次查验、一次放行”“一站式作业”等便利措施,全面提升贸易便利化水平,年内力争新增出口企业500家以上。坚持绿地投资、境外上市、国际并购、增资扩股“四位一体”吸引外资,实施“外商投资环境提升年”活动,落实外资企业“服务大使”“专家顾问小组”等制度,新设一批驻外经贸促进机构,推动已审批落地项目增资到账40亿元以上,新引进绿地投资项目40个以上。把园区作为开放发展的主战场,坚持“一园区一主导产业”,进一步明确各类开发区的产业方向。推进园区体制机制创新,探索“管委会+公司”运营模式,在高新区开展“亩均税收”领跑者机制试点,增强园区发展活力。

以企业家队伍建设为关键,夯实产业发展人才根基。事之成败,关键在人。深入开展向潍柴集团和谭旭光同志学习活动,着力打造本土企业家队伍,营造学习企业家、尊重企业家、服务企业家、爱护企业家的良好氛围。启动实施企业家培训工程,从龙头企业和“隐形冠军”企业选派100名以上企业家赴境外研修考察,从全市规模以上企业中选拔300名以上企业家在国内接受高端培训。完善新生代企业家培育体系,加强对青年创业者的培训指导。全面建立亲清新型政商关系,像爱护眼睛一样爱护企业家,像对待亲人一样对待企业家,与企业家风雨同舟、同心同向、同行同奋斗。大力弘扬企业家精神和工匠精神,引导广大企业家心无旁骛抓发展、诚信守法谋新篇,争当爱国敬业、自强不息、胸怀全局、无私奉献的典范。

以“一次办好”改革为抓手,营造一流发展环境。扎实推进优化营商环境10个专项行动,深入推进审批服务便民化。对接国家、省营商环境评价指标体系,建立完善我市营商环境评价标准。更大力度推进市级简政放权,深化扩权强县改革,积极推进“市县同权”。精准优化服务流程,全面抓好“五个一”集成服务改革,深入推进“互联网+政务服务”,进一步提升政务服务效能。加强事中事后监管,实现“双随机、一公开”监管常态化。深入推进信用体系建设,优化完善守信联合激励和失信联合惩戒制度。坚定不移促进民营经济发展,坚持“两个毫不动摇”“三个没有变”,全面贯彻省“支持民营经济高质量发展35条”“支持非公有制经济健康发展10条”,深入落实减税降费政策,让民营企业有更多获得感。持续开展营商环境突出问题专项整治,搭建以政策性担保为基础、风险缓释和补偿为支撑的民营企业融资保障体系,创造最适宜民营经济发展的外部环境。牢固树立“产业第一、企业为尊、纳税为荣、就业为重”理念,完善政企联谊机制,精准对接企业需求,以“店小二”的姿态当好服务员,做到无需不扰、有求必应。

(二)转变城市发展方式,建设人民满意城市。尊重城市发展规律,更好地统筹空间、规模、产业三大结构,统筹规划、建设、管理三大环节,统筹生产、生活、生态三大布局,推动城市发展由外延扩张向内涵提升转变,全面提升城市功能品质。

以科学精神做好城市规划。进一步增强规划的前瞻性、连续性、严肃性,用严谨求实的态度编制、完善、实施各类规划。编制新一轮城市总体规划(2020-2035年),优化“七城一体”发展规划,配套完善城市产业规划,推动中心城、卫星城、小城镇形成梯次、互补发展。中心城市全面落实“生态间隔、组团发展”理念,构建“城市中心、次中心、地区中心、社区中心”多层级布局体系。全力推进“多规合一”,搭建统一的信息管理平台,加快城市总体规划、土地利用规划、产业发展规划高度融合,把“一张蓝图干到底”的要求落到实处。深化中心城区总体城市设计,强化城市天际线、建筑风貌和色彩管控,更加注重传承历史文脉,加快形成独具特色的城市气质和城市风貌。

以人民满意为标准推进城市建设。坚持人民城市人民建,建好城市为人民。统筹用好城市空间,对棚户区改造、“散乱污”企业清理、拆违拆临腾出的土地,按照“保生态、补短板、强产业、控住宅”原则和“产城融合、职住平衡”理念,科学有序开发利用。中心城区重点抓好高铁新片区、站南广场片区建设,完善交通枢纽功能,加快引进培育城市产业,打造新的增长极。着力推进老市府金融商务区、齐鲁台湾城、坊茨小镇、食品博览园、VR智能小镇等一批高品质功能区建设。启动规划建设中央商务区,打造城市新地标,提升城市能级。积极稳妥推进棚改旧改,年内新启动棚改9966套。坚持安置房与商品房同标准建设,让拆迁居民充分享受棚改红利。坚持“房子是用来住的、不是用来炒的”定位,落实因城施策要求,合理有序供应土地,努力实现“稳地价、稳房价、稳预期、提品质”目标。着力改善城市生态,实施河道整治工程,整治城区河道40公里,恢复行洪功能,畅通城市“血管”,扮靓沿河风貌。实施雨污分流工程,加快雨水直排工程建设,完成城区29条街巷、107个小区雨污分流改造。落实海绵城市理念,让城市少些生硬、更接地气。

城市发展需要重大基础设施支撑拉动。要抓住国家推进基础设施领域补短板的重大机遇,主动对接京津冀、环渤海等国家战略,积极推进区域一体化特别是青潍一体化发展,全面提升基础设施联通连接水平。积极配合京沪高铁二通道天津至潍坊段现场勘测和可研报告编制,争取启动潍坊至临沂段前期工作。加快推进潍莱高铁、青潍城际铁路、潍烟高铁、德龙烟铁路扩能改造等项目建设。开工建设潍日高速连接线项目,完成济青高速改扩建工程,扎实做好济南至潍坊、潍坊至黄岛、潍坊至胶东机场、潍坊港疏港高速、莱州至董家口港等5条高速公路前期工作。积极推进机场迁建、轨道交通建设工作。加快潍坊港5万吨级航道建设,开展4个5万吨级通用泊位、1个2万吨级滚装泊位前期工作。加快乔官、官亭超高压工程和临朐抽水蓄能电站建设,完成峡山水库胶东地区调蓄战略水源地、黄水东调二期主体工程,打造山东半岛重要的能源水利枢纽。加快5G通讯等新一代信息基础设施建设。

用绣花功夫管理城市。城市管理,最是于细微处见功夫、见态度、见精神。积极推动智慧城市建设,加大WiFi广泛应用。围绕建设洁净舒适的城市,深入开展“清洁城市”专项行动,全面清理卫生死角,消除环境盲区。推动多层住宅加装电梯工作,加快推进小区物业管理全覆盖。围绕消除城市安全隐患,对城市河道泄洪、桥梁、供气输油管线进行定期“体检”、及时维修维护,确保安全稳定运行。全面启动地下管线普查,同步建立信息管理系统,保证地下管线坐标清晰、运行安全。实施好白浪河河堤修缮,完成白浪河危桥改造,加快解决城市积水内涝问题。围绕健全长效机制,坚持网格化、精细化、智慧化方向,全面落实路长制、门前三包等有效制度,推动城市管理转向城市治理。改造升级12319管理系统,搭建覆盖更广、效率更高的城市综合管理服务平台。围绕提升公用事业服务水平,推进供水厂深度工艺改造,提升污水、垃圾处理能力,强化供热、用气保障。年内开工建设中心城区自来水“提标扩容”工程和生活垃圾焚烧发电厂三期工程,完成沙窝污水处理厂恢复提升项目,解决城区污水和垃圾处理能力不足问题。完成城区57公里“汽改水”工程,实现高温水供热全覆盖。

实施城区次高压管线环网工程,推动滨海大型LNG储气站尽快落地建设。推动城市供热企业整合,让适当延长供热时间成为常态,确保市民温暖过冬。

今年,市政府将把城市“治堵”作为城市管理的突破口,顺应群众期盼,采取针对性措施,全面优化交通环境,下决心打好治堵攻坚战。实施道路畅通工程,年内完成潍县中路快速化改造,建成东、南两条外环路,完成4处交通环岛和45公里次支路网“微循环”改造,积极谋划立体化交通建设方案,建设一批城市停车场。全力实施打通“断头路”工程,用两年时间将中心城区主干道、次干道、支路和街巷的现存“断头路”全部打通。年内打通文化路、北宫街等10条主次干道“断头路”,完成10条下穿青银高速主次干道“卡脖路”改造,切实提高道路通行能力。优先发展公共交通,增加公交运力,优化公交线网,加大公交场站和公交专用线建设；完善城市慢行系统,持续推进公共自行车项目,优化共享单车运营,有序发展共享汽车,引导市民采用“步行+自行车+公交”方式出行。启动实施智慧交通综合提升工程,综合运用大数据、人工智能、云计算等技术手段,推进数据资源互联互通、共享共用,大幅提升城市交通一体化、智能化管控水平。力争用两年时间,中心城市60%以上主干道实现绿波控制,其余主次干道实现自适应控制,市区高峰时段行车速度提升20%以上。

(三)大力实施乡村振兴战略,创新提升“三个模式”。认真落实习近平总书记推进乡村“五大振兴”的要求,进一步提炼升华“诸城模式”“潍坊模式”“寿光模式”,持续推进农业更强、农村更美、农民更富。

抓好两大国家级平台建设。国务院批准设立的国家农业开放发展综合试验区、省部共建的全国蔬菜质量标准中心,都是全国唯一的现代农业发展平台,是实施乡村振兴战略的重要载体,必须用心用力抓好。国家农业开放发展综合试验区建设,坚持世界眼光、国际标准,立足山东优势、潍坊特色,围绕28项试验内容和11条支持政策,统筹“引进来”和“走出去”,加强体制机制创新,广泛集聚产业、技术、人才、信息、资金等资源要素,加快核心区“五个中心”建设,加强与保税区北区联动,促进辐射区各类急需项目平台建设,确保“一年全面起势、三年大见成效、五年形成样板”,把综试区打造成全国农业开放发展引领区、农业科技创新先行区、农村一二三产业融合发展示范区,基本形成农业开放发展新格局,率先实现农业农村现代化。年内启动国际院士谷、国际食品展示交易中心暨电商产业园建设,建成运营国家现代农业产业园技术集成示范区一期、国际种业研发集聚区一期工程。全国蔬菜质量标准中心建设,发挥政府引导和市场主体作用,协同国家蔬菜标准创新基地等已有平台,用好数量庞大、遍布全国的蔬菜专业人才队伍,用两年时间建立覆盖蔬菜全产业链的标准化体系,形成可以全国推广应用的标准化模式。年内编制实施设施蔬菜产品全产业链标准体系规划,全面推进蔬菜全产业链标准集成和研发中心等“四个中心”建设,建成1.7万亩示范推广基地和蔬菜信息大数据服务中心。

提升现代农业发展水平。加快发展蔬菜、畜牧、苗木、花卉四大优势产业,提升质量标准,增强带动能力。以培育发展农业“新六产”为主攻方向,深度推进农村一二三产业融合,集中打造10家领军型农业产业化联合体,建设一批农业“新六产”示范主体。加快智慧农业发展,探索建立涵盖农业、林业、畜牧、渔业的现代农业大数据应用工程。加强为农服务体系建设,开展农民专业合作社整合提升行动,新创建市级示范社、示范场各60家,国家级示范社数量达到55家以上。做大做强3家百亿级、3家50亿级、8家30亿级农业龙头企业。建成运营北京大学现代农业研究院。健全完善鼓励城市工商资本下乡政策体系,年内推动城市工商资本下乡突破30亿元。扎实推进省市两级田园综合体试点项目建设。推进“潍坊农品”品牌建设,重点培育寿光蔬菜、潍县萝卜、青州银瓜等特色农产品品牌,新认证绿色产品、有机农产品50个以上。实施“优质粮食工程”,保障粮食安全和主要农产品有效供给。

夯实农业农村人才基础。完善鼓励农民返乡创业政策,全面支持“雁归”群体和外出农民工回乡创业就业。实施好农业科技“展翅行动”,健全新型职业农民培养体系,实施好农业高端人才聚集、乡村实用人才培育、人才“上山下乡”等工程,年内培训基层农技人员800名以上,培育新型职业农民3000人。搭建创新创业平台,开展新型职业农民创业大赛,引导各类人才向农村基层集聚。

建设生态宜居美丽家园。深入推进农村人居环境整治三年行动,积极探索农村污水治理模式,开展农村生活垃圾分类试点,建立农村改厕后续管护长效机制,完成涉农街道办事处改厕17万户,全面完成现有农村存量危房改造。实施农村饮水安全提标达标两年攻坚行动,年内新建改造规模化水厂3处,完成80个村级工程巩固提升任务,实施2处水厂信息化提升工程。持续推进“四好农村路”建设,新建改造农村公路1350公里。统筹做好南部生态涵养区保护开发。完成国土绿化15万亩。积极推动乡村旅游与农业、林业、渔业、畜牧等产业融合发展,年内打造60个A级景区村庄,形成10个以上乡村旅游集群片区。坚持既塑形又铸魂,大力传承发展农村优秀文化,深入实施爱诚孝仁“四德工程”,扎实开展名镇名村志书编修试点,扶持建设“农家书屋”或图书室,规划建设一批村史馆、乡村记忆博物馆和乡村红色教育基地,让人们能够“看得见山水、记得住乡愁”。

构建坚强有力的农村组织体系。健全自治、法治、德治相结合治理模式,完善“四议两公开”决策机制、村民自治机制、民主监督机制,建设和谐乡村。基本完成农村集体产权制度改革任务,健全完善农村土地“三权”分置制度。加强农村法治建设,集中整治农村黑恶霸痞犯罪、封建迷信、非法宗教等突出问题。强化平安乡村建设,持续推进“雪亮工程”,加快形成共建共治共享的乡村治理新格局。

(四)坚决打好三大攻坚战,夯实高质量发展基础。提高政治站位,强化底线思维,落实保障措施,压实相关责任,加快形成标本兼治、系统发力的良性机制。

切实防控化解重大风险。将防控金融风险放在突出位置,坚持早识别、早预警、早处置,建立多层次风险防控化解机制。严厉打击恶意逃废银行债务行为,加大不良贷款核销力度,充分发挥银行业债权委员会、资产管理公司、融资性担保机构、应急转贷基金、过桥基金、并购基金等作用,确保不良贷款率控制在合理范围。加强国有企业资产负债约束,强化企业投融资和财务监督,市级国有投融资类企业重大投资实行报批制。推进综合执法监管,严厉打击各类违法违规金融活动。坚决遏制政府隐性债务增长,有序消化债务存量,规范政府举债融资行为,杜绝各类违法违规举债,确保政府显性债务率保持在警戒线以下,综合债务率大幅度下降。全面抓好安全生产,加快建设风险分级管控和隐患排查治理双重预防体系,开展运行评估和专项执法检查；实施城市安全设施改造,创建“安全发展示范城市”。加强应急救援队伍建设,补齐消防安全设施短板,配备一台101米登高平台消防车。健全综合防灾减灾救灾体系,全面提升抵御自然灾害的综合防范能力。坚持发展“枫桥经验”,完善多元纠纷解决机制,依法及时就地化解各类矛盾问题。织密织牢“五张网”,巩固立体化、信息化社会治安防控体系。有效防范和化解移动互联网安全风险。不断深化扫黑除恶专项斗争,全面整改中央扫黑除恶巡视反馈问题。坚决打赢新时代禁毒人民战争,建设公安强制隔离戒毒所和特殊监区。严厉打击侵财犯罪和电信网络诈骗犯罪,保障群众生命财产安全。

巩固提升脱贫攻坚成果。坚持精准扶贫、精准脱贫方略,注重标准质量,聚焦“两不愁三保障”目标,严格识别退出标准和程序,坚决杜绝数字脱贫、算账脱贫、虚假脱贫。坚持开发式扶贫与保障式扶贫相结合,创新产业扶贫方式,支持产业扶贫重点项目建设,培育扶贫龙头企业。加快推进就业创业扶贫,加大贫困人口技能培训,帮助贫困户依靠自身脱贫致富。加强扶贫资金监管使用,严格扶贫项目管理运营,提升产业项目收益率,完善收益分配机制。落实“311”民生公共政策,推广贫困人口智慧医护养老模式,减少贫困人口大项支出。建立稳定脱贫长效机制,完善内外联动、行业扶贫和定点扶贫等工作机制,加快构建政府主导、部门发力、全社会共同参与的大扶贫格局。

全面提升污染防治成效。良好生态环境是最公平的公共产品,也是最普惠的民生福祉。要深入践行习近平生态文明思想,坚持“绿水青山就是金山银山”,全面落实“四减四增”任务,以环境容量倒逼绿色发展、倒逼淘汰落后产能、倒逼产业转型升级。开展生态环境问题整治大会战,集中解决一批环境领域突出问题。严格按标准要求抓好中央、省环保督察及“回头看”反馈问题整改,确保整改见底到位。严厉打击环境领域违法犯罪行为,严肃问责“表面整改”“假装整改”“敷衍整改”问题,扎实推进蓝天、碧水、净土三大保卫战。持续加强大气污染防治。全面实施第四时段污染源达标提标改造。年内中心城区6家企业全部完成搬迁或关停。淘汰13台中心城区燃煤锅炉、21台10万千瓦以下抽凝机组。以县市区主要污染物月均浓度和空气质量优良天数变化情况为考核指标,进一步完善空气质量补偿机制。年内二氧化硫、氮氧化物排放总量分别较2015年削减23.2%和23.6%。重污染天数持续下降,PM2.5、PM10浓度同比分别改善5%和10%左右,空气质量优良天数比例达到60%以上。突出抓好水污染防治,市控及以上重点河流全面消除劣五类水体并稳定达标,化学需氧量、氨氮排放量分别较2015年削减10.1%和13.4%。开展“深化清违整治、构建无违河湖”专项行动,进一步深化河长制、湖长制、湾长制。严守南部山区生态屏障,按照主体功能区定位,加快编制“三线一单”,严格限定产业准入类别,明确禁止和限制发展的行业、生产工艺和产业目录,进一步加强源头控制。开展环渤海排污口排查整治,全面提高涉海陆源排放标准,强化北部沿海滩涂湿地和海域生态保护修复,坚决打好渤海综合治理攻坚战。以入海河流水质达标倒逼流域污染整治,启动潍河、弥河、虞河、白浪河等4条市级河流生态综合治理,全力争取主要指标达到三类水标准。提升污水处理能力,新建扩建污水处理项目8个,提标改造污水厂5个。加强土壤污染管控和修复。抓好农业面源污染防治,农药使用量保持零增长,化肥使用量减少1%以上,畜禽粪污综合利用率达到80%以上。加强危险废物监管能力建设,建立危险废物产生、收集、贮存、利用和处置等全过程监管体系。

(五)夺取灾后重建全面胜利,向全市人民交上满意答卷。全面落实省、市8个方面30条政策,深入实施《潍坊市灾后重建总体方案》和6个专项方案。继续发挥联系卡作用,精准对接受灾群众生产生活需求,细化帮扶措施。把重建质量作为灾后重建的“生命线”,严格准入门槛,加强全过程监管,落实终身责任制,切实把灾后重建各项工程打造成优质工程、民心工程。严格按照既定的时间节点要求,倒排工期,挂图作战,加快工作进度。4月底前,完成大棚重建任务；5月底前,完成水库、拦河闸坝、河道、农田排涝等水利设施重建任务；7月底前,完成农村公路、桥梁、涵洞重建任务；8月底前,全市15个整村迁建项目全部竣工。

(六)大力保障和改善民生,持续增强群众的获得感、幸福感、安全感。民生是最大的政治。“以人民为中心”是各项工作的根本遵循。要始终牢记“全心全意为人民服务”的根本宗旨,牢固树立人民立场,自觉做到发展为了人民、发展依靠人民、发展成果由人民共享,不断完善公共服务,提高民生保障水平。

促进高质量就业。把稳就业摆在突出位置,全方位优化就业服务,统筹推进高校毕业生、返乡农民工等重点群体就业,实现城镇新增就业10万人。深化“185”创业行动,加快建设互联网新媒体创业大学,持续推动创业带动就业。落实援企稳岗政策措施,完善劳动人事争议调解仲裁制度,促进劳动关系和谐稳定。

优先发展教育事业。深化教育体制机制改革,努力建设现代化教育强市。加快学前教育普惠发展,统筹推进义务教育优质均衡发展,深化普通高中综合改革。全力打造职业教育创新发展示范区,加强职业教育基础能力建设,支持市属高等院校创建优质校,加快创建山东海洋技术大学。加强新时代教师队伍建设,实施校长治校育人能力提升工程。完善学校安全促进体系。

加快建设健康潍坊。深化医药卫生体制改革,推进“四医”联动,落实基层医疗卫生机构“一类保障、二类管理”制度,加快分级诊疗和医联体建设。创建国家中医药综合改革示范区,建设中医药强市。实施“健康潍坊人”行动计划,探索建立居民健康指标体系。开展全国社会心理服务体系建设试点。创建全省首个慢性病综合防控示范市。提升计生服务管理水平。深入推进餐饮质量安全提升工程,深化药品医疗器械审评审批制度改革。做好国家卫生城市复审迎查工作。办好第二十届潍坊市运动会。

持续强化社会保障和社会治理。深入实施全民参保计划,完善大病保险制度,深化企业养老保险制度改革,全面落实被征地农民养老保险补贴政策,推进新开工工程建设项目工伤保险全覆盖。加快推进全国居家和社区养老服务改革试点,建设智慧养老管理服务平台。健全完善社会救助体系,不断提高救助服务水平。深化普法依法治理和公共法律服务体系建设。支持工会、共青团、妇联等群团组织参与社会治理。加强退役军人服务保障体系建设,扎实做好退役军人优抚安置等工作。加强国防教育、国防动员、双拥共建、民兵预备役、人民防空等工作,全力支持国防和军队现代化建设,营造尊崇军人、景仰英雄的浓厚氛围。

繁荣发展文化事业。扎实做好新时代文明实践中心建设。加快推进县域公共文化场馆“总分馆”制建设。积极创建联合国手工艺与民间艺术之都。开展庆祝建国70周年主题文化活动。深入实施文化惠民工程,努力提高公共文化服务水平。扎实推进国家级潍水文化生态保护实验区建设,办好第三届文化惠民消费季、第四届市民文化节等活动。

悠悠万事,民生为大。市政府今年将适当压减市级一般性支出,适当提高民生支出市级财政分担比例(市级分担由原来不足10%提高到30%),积极引导社会资本参与,把群众最需要、最关切的事情一件一件解决好,年内重点办好32件民生实事:

1.实施校园建设三年攻坚行动,坚持财政投入与市场化运作相结合,投资78.5亿元,年内新建改扩建优质中小学校81所、标准化普惠性幼儿园140处。

2.投入1.2亿元,将公办义务教育学校班级管理团队激励机制经费财政负担部分,由每月400元提高到500元。

3.投入7520万元,为公办义务教育学校教师按生均每年100元核增绩效工资增量,进一步提高一线教师待遇。

4.投入5190万元,全面推行小学“弹性上学”与课后延时服务,打造素质教育第二课堂,让家长的“操心事”变成“暖心事”。

5.投入2亿元,将普通高中生均公用经费由1100元提高到1300元,残疾学生生均公用经费由6000元提高到8000元,让教育保障政策惠及更多家庭。

6.多方筹集资金21.28亿元,启动北辰现代教育示范区建设,优化中心城区教育资源配置,更好满足居民对现代化优质教育的需求。

7.投入8300万元,将人均基本公共卫生服务经费标准由55元提高到64元,筑牢公共卫生屏障。

8.投入4704万元,为孕妇免费提供产前筛查,降低出生缺陷发生率。

9.引进“关爱新生命”公益项目,投入9780万元,为全市新生儿免费进行48种遗传代谢疾病筛查；为贫困家庭3岁以下婴幼儿免费提供奶粉,其他家庭价格优惠比例由50%提高到70%,有效减轻家庭育儿负担。

10.实施“全民艾健康”行动计划,投入2000万元引导性资金,推广峡山区太保庄“热敏灸小镇”经验,各县市区、市属开发区至少选择一个村(社区)开展试点,所有镇(街道)卫生院、社区卫生服务中心、养老院配置艾灸设备、开展艾灸活动,有效预防治疗常见病、慢性病。

11.投入5527万元,为70岁以上老年人免费接种四价流感疫苗,降低流感对老年人群的健康影响。

12.投入2300万元,为1岁儿童免费接种水痘疫苗,有效预防高发病率、高传染性水痘急性传染病。

13.投入4000万元,为全市适龄(35-64岁)妇女提供“两癌”免费筛查(五年一轮),筑牢妇女健康“防护墙”。

14.投入1.52亿元,为6种严重精神障碍患者提供免费治疗。

15.深入推进“食安潍坊”品牌创建,每年投入1320万元,购买“透明快检”服务,提高问题食品发现率,严把食用农产品入市进店关口,捍卫百姓“舌尖上的安全”。

16.每年投入2952万元,利用三年时间,建成328处标准化社区居民服务中心,打造社区居民安居乐业的温馨之家。

17.积极营造尊老敬老社会氛围,投入3.23亿元,为80周岁以上老年人提高高龄补贴标准(对80-89岁老年人每人每月补贴100元,将90-99岁老年人高龄补贴由每人每月60元提高到150元,将百岁老年人长寿补贴由每人每月600元提高到1000元),让全市老年人更多享受改革发展成果。

18.投入4.15亿元,全面提高城乡低保和特困人员供养及护理标准,提高保障水平,让他们生活得更有尊严、更有奔头。

19.投入1.37亿元,对困难残疾人生活补贴和重度残疾人护理补贴提标扩面,全力保障其基本生活。

20.投入623万元,将在校残疾学生助学补助标准提高30%,贫困残疾人子女学生按照残疾学生补助标准50%执行,让他们坚定成才信心,顺利完成学业,追求美好生活。

21.完善残疾儿童康复训练救助制度,投入1390万元,分类提高残疾儿童康复训练救助标准,让他们更加勇敢地迎接生活挑战,为实现人生梦想而奋斗。

22.着力提高老年人养老保障水平,新投入1.02亿元,将65-74岁、75岁以上居民基础养老金由每人每月118元分别提高到123元和128元。

23.投入2100万元,将缴费困难居民基本养老保险费政府代缴标准由每人每年100元提高到300元,让他们享受与广大居民同等的养老保险待遇。

24.投入850万元,实施全民健身设施全覆盖工程,新建更新行政村(社区)健身工程480处,在中心城区新建55处全民健身设施和一批球类运动场地,完善“10分钟健身圈”。

25.投入7933万元,改造中心城区“微空间”,新建续建111处口袋公园,拓展绿色活动空间,为市民提供更多休憩之地。

26.投入1500万元,在中心城区新建100座公厕,方便市民生活,提升城市品位。

27.投入1000万元,为符合参保条件的环卫工人办理意外伤害保险。

28.投入6700万元,对中心城区提前和延长供热居民采暖进行补贴,让群众既暖身又暖心。

29.投入6400万元,对农村新型社区和小城镇进行优化提升,启动涉农街道家庭无害化卫生厕所改造,不断改善农村居民生活环境。

30.优化提升中心城区慢行交通,投入1000万元,新建公共自行车站点60处,新投放公共自行车1000辆,规划建设200处共享单车停放区,进一步方便居民绿色低碳出行。

31.坚持公交优先,投入1.4亿元,支持购置新能源和清洁能源公交车,进一步织密市区公交网,让市民享受更加密集、便捷的公交服务。

32.采取政府与社会资本合作模式,利用两年时间投资3.8亿元,启动智慧交通建设工程,对中心城区原有交通管理系统进行整合提升,建设智慧交管平台和城市交通大脑,实现数据共享、智能应用。年内完成投资1.5亿元,加快实现城市交通综合一体化智能管控,进一步提升交通运行效率。

三、全面加强政府自身建设

“大道至简,实干为要。”作为人民政府,必须强化落实导向,树牢为民情怀,以自身的勇担当、善作为,赢得人民群众的信赖和支持,努力建设人民满意的政府。

扎实推进“工作落实年”活动。“一分部署,九分落实。”要紧扣“转观念、正风纪、夯基础、提质量、促发展”主题,全面开展大讨论、大调研、大整改、大推动,以“钉钉子”精神推动各项工作落实。细化工作责任,完善推进机制,实施项目化管理,确保上级决策部署和市委工作安排落地生根、开花结果。严格落实中央关于改进工作作风、密切联系群众规定,坚决防止“四风”反弹回潮。深入整治形式主义、官僚主义,大幅减少文件会议,规范督查检查,让基层有更多的时间和精力抓落实。大兴调查研究之风,察实情、出实招、办实事,把调查研究贯穿于决策和执行全过程。牢固树立过紧日子思想,坚持勤俭办一切事业。完善激励机制和容错纠错机制,为担当者担当,让实干者实惠,营造积极担当作为、激情干事创业的良好生态。

大力提升行政效能。“取法其上,得乎其中。”要坚持高起点、高标准,加快转变政府职能,充分发挥市场在资源配置中的决定性作用,更好发挥政府作用。加强部门协作配合,汇聚推动工作的强大合力。借势政府机构改革,着力构建权责分明、运转高效、充满活力的扁平化体制机制。深入推进“智慧潍坊”建设,强化大数据辅助政府决策治理的能力。加快一体化在线政务服务平台建设,推进政务服务事项跨地区、跨部门、跨层级协同办理。实行部门联合监管,做到“一次检查、全面体检”。发挥好政务服务热线作用,强化热线大数据精准分析,提升政府工作的针对性和实效性。切实提高政府决策的透明度、开放度和市民参与度,落实市民代表列席市政府常务会议制度,努力把政府决策过程变成汇聚民意、集中民智、凝聚民心的过程。

加快建设法治政府。“法者,治之端也。”要大力弘扬宪法精神、增强宪法意识,坚持依宪施政、依法行政,把政府各项工作全面纳入法治轨道。主动接受法律监督、监察监督、审计监督、民主监督、社会监督、舆论监督,积极配合监察机关工作。高质量办好人大代表议案建议和政协委员提案。严格执行地方性法规和政府规章制定程序,继续加强重点领域立法。推动市县两级行政审批服务局依法实施相对集中行政许可权。全面落实行政执法公示、执法全过程记录、重大执法决定法制审核“三项制度”,推行行政裁量权基准制度。加快行政处罚和行政强制权力事项上网运行。全面深化政务公开工作。加强行政复议规范化建设。依法依规做好第四次经济普查工作。

持续增强专业素养。“事必以专,方显其功。”要大力培养政府工作人员的专业精神,以专业思维、专业方法提高履职能力。定期开展业务知识培训和岗位比武活动。建立健全以专业知识和专业能力为导向的考核评价机制,完善差异评价和正向激励措施,推动政府人员学习专业知识、研究本职工作、提升业务能力。进一步增强政府工作人员在学习、政治领导、改革创新、科学发展、依法行政、群众工作、狠抓落实、驾驭风险等八个方面的本领,引进一批高层次专业人才,努力建设讲政治、敢担当、有激情、重诚信、守底线的高素质干部队伍。

全面加强廉政建设。“善禁者,先禁其身而后人。”要严格落实全面从严治党主体责任,认真履行党风廉政建设“一岗双责”,扎实推进政府系统党风廉政建设向纵深发展。深入推进反腐败斗争,坚持无禁区、全覆盖、零容忍,始终保持惩治腐败高压态势,巩固发展反腐败斗争压倒性胜利。严格执行财经纪律和议事规范。坚决整治群众身边腐败,开展扶贫领域腐败和作风问题专项治理。扎实做好巡视巡察发现问题整改。聚焦重点领域,严管公共权力、公共资金、公共资产、公共工程和公共资源交易。

各位代表,新时代是奋斗者的时代。习近平总书记强调,伟大梦想不是等得来、喊得来的,而是拼出来、干出来的。让我们更加紧密地团结在以习近平同志为核心的党中央周围,在习近平新时代中国特色社会主义思想指引下,在省委、省政府和市委坚强领导下,撸起袖子加油干,扑下身子抓落实,为决胜全面小康、建设现代化强市而努力奋斗!
